# β-Cyclodextrin Inclusion Complexes of 20-Hydroxyecdysone Derivatives: Synthesis, NMR Characterization, and In Vitro/In Vivo Evaluation of Antioxidant, Hepatoprotective, and Antimicrobial Activities

**DOI:** 10.3390/ph19060885

**Published:** 2026-06-02

**Authors:** Borash Tuleuov, Aizhan Zeinuldina, Aidana Bazarkhankyzy, Aizhan Kozhanova, Bakhtiyar Temirgaziyev, Saniya Dyussekeyeva, Lyazzat Abulyaissova, Nurgul Askarova, Aliya Temirbekova, Zhanar Tekebayeva, Ardak Sapiyeva, Sergazy Adekenov

**Affiliations:** 1 Laboratory of Chemistry of Steroid Compounds, JSC “Scientific and Production Center “Phytochemistry”, Karaganda 100009, Kazakhstannurs-djoni@mail.ru (A.K.);; 2 Department of General and Biological Chemistry, Astana Medical University, Astana 010000, Kazakhstan; bazarkhankyzy.a@gmail.com (A.B.); serykpaeva@gmail.com (S.D.); nurgulasskar@gmail.com (N.A.); 3 Department of Physical and Analytical Chemistry, Buketov Karaganda University, Karaganda 100028, Kazakhstan; 4 Laboratory of Microbiology, Republican Collection of Microorganisms LPP, Astana 010000, Kazakhstan

**Keywords:** phytoecdysteroids, 20-hydroxyecdysone, cyclodextrins, hepatoprotective activity, DPPH assay, FRAP assay, antimicrobial activity

## Abstract

**Background/Objectives**: For the first time, water-soluble encapsulated forms of 20-hydroxyecdysone were synthesized as clathrate complexes with β-cyclodextrin. The derivatives included a ketoxime form (20-NOH), obtained by selective oximation of the C-6 carbonyl, and a triacetate form (3Ac-20E), obtained by regioselective acetylation of the C-2, C-3, and C-22 hydroxyl groups. **Methods**: The chemical structures of the obtained compounds were confirmed using spectroscopic methods, including ^1^H and ^13^C NMR. The Z-configuration of the oxime in 20-NOH was established by the diagnostic upfield shift in the C-5 resonance (δ 36.0 ppm) relative to the parent compound (δ 51.4 ppm). **Results**: The antioxidant activity was evaluated using FRAP and DPPH assays. The β-cyclodextrin complexes were shown to exhibit significantly higher radical scavenging activity, with IC_50_ values of <0.05 mg/mL compared to 0.24 mg/mL for the parent 20-hydroxyecdysone (20E). In the FRAP assay, the complexes demonstrated enhanced reducing capacity, with values of 3.452 ± 0.520 AAE/mL for the ketoxime form (20-NOH·β-CD) at a concentration of 0.75 mg/mL and 3.810 ± 0.279 AAE/mL for the acetate form (3Ac-20E·β-CD) at the same concentration, whereas the parent compound did not exceed 0.46 AAE/mL. In both in vitro and in vivo experiments, it was established that the investigated complexes demonstrated hepatoprotective properties, as evidenced by the normalization of biochemical parameters as well as the restoration of liver structure according to ultrasound and histological studies. The ketoxime-derived complex (20-NOH·β-CD) at a dose of 25 mg/kg demonstrates the greatest reduction in transaminase activity and MDA levels, indicating its high efficacy and the presence of an optimal therapeutic dose. In addition, the encapsulated forms demonstrated moderate antifungal activity against *Candida albicans* and bactericidal activity against four bacterial strains (MIC >10 mg/mL). **Conclusions**: The obtained results indicate that β-cyclodextrin complexes significantly enhance the biological activity of 20E and highlight the potential of these compounds for pharmaceutical applications.

## 1. Introduction

An innovative approach to the development of a new generation of pharmaceuticals is the design of phytopreparations that enhance the body’s resistance and ensure the normal functioning of its organs and systems. In this context, plant-derived polyhydroxylated sterols—phytoecdysteroids—are of particular interest, as they do not exert hormonal effects in mammals.

One of the most extensively studied phytoecdysteroids is 20-hydroxyecdysone (20E, or ecdysterone), which is included in tonic, anabolic, and adaptogenic phytopreparations such as “Ecdysten” and “Ecdyphyt” [1,2,3,4]. The wide range of pharmacological properties of 20-hydroxyecdysone enables its use both as a single agent and as part of various composites and conjugates. Currently, there has been significant progress in the study of 20-hydroxyecdysone, with its pharmacological effects in various pathologies and its regulatory influence on metabolic processes being actively investigated [5,6,7,8].

Modern trends in the development of new phytopreparations are focused on creating water-soluble compositions with tailored physicochemical properties and enhanced bioavailability. One of the approaches to obtain a water-soluble form of 20-hydroxyecdysone is the synthesis of its nanosized clathrate complexes with clathrate-forming agents, including cyclodextrins. The encapsulated water-soluble form of 20-hydroxyecdysone synthesized in this way improves its physicochemical properties, leading to increased absorption, enhanced bioavailability, and ultimately allowing for a reduction in therapeutic doses [9,10,11].

Currently, considerable attention is focused on investigating their biological activity, including antioxidant, hepatoprotective, and antimicrobial properties [12,13].

The first report on the hepatoprotective activity of 20-hydroxyecdysone was provided by Badalyants et al. [14], where ecdysterone was shown to prevent adverse changes in phospholipid metabolism and to maintain high levels of glycogen synthesis and protein synthetic function in the liver. These effects underlie its hepatoprotective activity in acute heliotrine-induced intoxication.

Ramazanov et al. [15] concluded that *Stachys hissarica* is highly promising as a source for the development of new effective hepatoprotective agents. Biological studies in male rats demonstrated that a combined preparation containing 20E, polypodin B, 2-deoxy-20-hydroxyecdysone, integristerone A, and 2-deoxy-α-ecdysone exhibits pronounced hepatoprotective activity under conditions of toxic liver injury induced by triple administration of indomethacin at a dose of 20 mg/kg and surpasses the well-known reference drug Liv 52.

Furthermore, Syrov et al. [16], the same authors investigated a phytocomposition consisting of soy lecithin, glycyrrhizic acid, lycopene, and ecdysterone (commonly referred to as Hepalipin), which significantly prevents the development of cytolytic–cholestatic liver injury. This composition was shown to support protein and glycogen synthesis in the liver, reduce fatty degeneration, suppress disturbances in pigment metabolism, and exhibit antioxidant activity when administered to experimental animals with developing chronic toxic hepatitis induced by CCl_4_.

Zador [17] also reports that, in animal studies, 20E exhibits anabolic, antioxidant, antidiabetic, anti-obesity, cardioprotective, neuroprotective, hepatoprotective, and other biological effects.

The structural complexity of 20-hydroxyecdysone, bearing six hydroxyl groups, presents a fundamental challenge for selective chemical modification: the polyfunctionality of the molecule inevitably leads to the formation of complex, difficult-to-separate reaction mixtures under standard conditions. Moreover, the pronounced hydrophilicity of the polyol framework paradoxically results in poor aqueous solubility at physiologically relevant concentrations, limiting the bioavailability of the parent compound. These constraints directed our synthetic strategy toward well-precedented chemical transformations of the ecdysteroid scaffold—selective oximation and regioselective acetylation—followed by supramolecular encapsulation with β-cyclodextrin. In the present study, 20-hydroxyecdysone and two of its chemically modified forms were selected as the objects of investigation: the inclusion complex of 2,3,22-triacetyl-20-hydroxyecdysone with β-cyclodextrin (3Ac-20E·β-CD) and the inclusion complex of the ketoxime derivative with β-cyclodextrin (20-NOH·β-CD). This approach simultaneously addressed two objectives: the generation of structurally defined derivatives with altered functional group profiles, and the improvement in aqueous solubility through host–guest complexation. These compounds were selected for a comparative evaluation of the biological activity of the parent phytoecdysteroid and its water-soluble encapsulated derivatives, based on the central hypothesis that β-cyclodextrin complexation, by enhancing aqueous solubility and bioavailability, would significantly potentiate the antioxidant and hepatoprotective properties of 20E. Accordingly, the primary aim of this study was to synthesize and characterize novel β-cyclodextrin inclusion complexes of 20E derivatives and to evaluate their antioxidant, hepatoprotective, and antimicrobial activities in vitro and in vivo, thereby assessing the therapeutic potential of encapsulation as a strategy for improving the biopharmaceutical properties of phytoecdysteroids.

## 2. Results and Discussion

### 2.1. Chemistry

#### 2.1.1. Isolation of 20-Hydroxyecdysone

**Figure 1:**
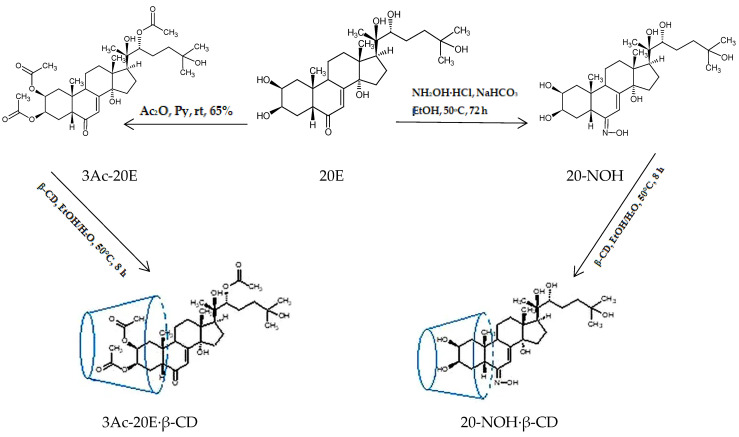
Synthesis of 3Ac-20E, 20-NOH and their inclusion complexes with β-cyclodextrin.

20-Hydroxyecdysone was isolated from the aerial parts of *Silene wolgensis*, a plant species previously reported as a rich source of phytoecdysteroids. The isolation yielded 3.0 g of 20E with a purity of 98.2% from 2.0 kg of dry plant material, corresponding to a yield of approximately 0.15% (*w*/*w*), which is consistent with published values for ecdysteroid content in *Silene* species (0.1–0.3% dry weight). The co-isolated minor phytoecdysteroid 2-deoxyecdysone, obtained in fractions of lower polarity, differs from 20E by the absence of the C-2 hydroxyl group, confirming the structural selectivity of the chromatographic separation. The chemical identity of 20E was confirmed by melting point (mp 236–238 °C), optical rotation ([α]D^2^° + 66.0), and characteristic UV absorption at λmax 244 nm (log ε 4.10), attributable to the α,β-unsaturated ketone (enone) chromophore of the ecdysteroid skeleton (C-6 carbon-yl conjugated with the C-7,8 double bond). IR absorption at 3320 cm^−1^ confirmed the presence of multiple hydroxyl groups. The structural formula of 20E, highlighting the six hydroxyl groups and the enone system, is presented in Figure 1. The relatively low aqueous solubility of 20E, inherent to its steroidal scaffold, provided the principal motivation for chemical derivatization and subsequent cyclodextrin encapsulation in the present work.

#### 2.1.2. Synthesis of 2,3,22-Triacetyl-20-Hydroxyecdysone and Its Inclusion Complex with β-Cyclodextrin

The selective acetylation of 20E with acetic anhydride in anhydrous pyridine afforded two products: the 2,3,22-triacetate (3Ac-20E) as the major product in 65% yield, and the 2,3,22,25-tetraacetate as a minor product in 29% yield. The preferential formation of the triacetate reflects the well-established reactivity hierarchy of hydroxyl groups in ecdysteroids: the secondary allylic C-2-OH and equatorial C-3-OH, as well as the secondary C-22-OH in the side chain, are significantly more reactive toward acetylation than the sterically hindered tertiary C-25-OH, which is flanked by two methyl groups (C-26 and C-27). This regioselectivity pattern is consistent with that reported for acetylation of other ecdysteroids with similar polyol side chains. The selective protection of C-2, C-3, and C-22 hydroxyl groups in 3Ac-20E is chemically significant: it eliminates the most reactive OH groups while preserving the C-14-OH and C-25-OH, thereby modifying the hydrogen-bonding profile of the molecule without altering the enone chromophore (C-6/C-7=C-8). This selective modification was specifically chosen to investigate how changes in hydroxyl group accessibility affect antioxidant activity and cyclodextrin encapsulation efficiency relative to the parent compound. The formation of the β-cyclodextrin inclusion complex of 3Ac-20E was confirmed by NMR spectroscopy, IR analysis (characteristic shifts in β-CD bands at 947 and 1029 cm^−1^), UV (λmax 245 nm), and the elevated decomposition melting point (267 °C), the latter indicating a thermally stabilized host–guest assembly.

#### 2.1.3. Synthesis of Ketoxime Derivative of 20-Hydroxyecdysone and Its Inclusion Complex

The oximation of 20E with hydroxylamine proceeded with high regioselectivity, targeting the C-6 carbonyl of the enone system in preference to the C-20 position, and yielding the ketoxime derivative 20-NOH. Although the C-6 carbonyl is part of an α,β-unsaturated ketone system conjugated with the C-7,8 double bond, the reaction reached completion under forcing conditions (50 °C, 72 h). This transformation is unambiguously confirmed by ^13^C NMR spectroscopy: the C-6 ketone resonance at δ 203.4 ppm disappears entirely, with concomitant appearance of a new C=N–OH signal at δ 154.0 ppm, while the C-20 resonance remains unchanged at δ 76.8 ppm.

A key diagnostic indicator is the marked upfield shift in the C-5 resonance from δ 51.4 ppm (in 20E) to δ 36.0 ppm (in 20-NOH), which not only confirms oxime formation at the adjacent C-6 position but also provides definitive evidence for the Z-configuration of the oxime. The replacement of the ketone with an N–OH group significantly modulates the hydrogen-bonding profile and polarity of the molecule.

The β-cyclodextrin inclusion complex of 20-NOH·β-CD was subsequently obtained under moderate conditions (40–50 °C, 8 h) and isolated as a crystalline precipitate. The formation of this stable host–guest assembly, driven primarily by hydrophobic interactions between the ecdysteroid framework and the cyclodextrin cavity, is further corroborated by the NMR and computational data presented here in.

The synthesis of 3Ac-20E, 20-NOH, and their inclusion complexes with β-cyclodextrin is illustrated in Figure 1.

### 2.2. Spectroscopic Analysis

#### NMR Spectroscopic Study of Structure and Inclusion Complex Formation

^1^H and ^13^C NMR Data of 20-Hydroxyecdysone (20E)

The NMR spectra of 20-hydroxyecdysone were recorded in deuterated chloroform (CDCl_3_), due to the good solubility of the parent compound in this solvent and the ability to obtain high-resolution signals without significant line broadening.

^1^H NMR (400 MHz, CDCl_3_, δ, ppm, J/Hz): 5.63 (^1^H, d, J = 2.6, H-7), 3.76 (^1^H, br s, H-3), 3.60 (^1^H, ddd, J = 11.9, 4.3, 3.1, H-2), 3.12 (^1^H, dd, J = 10.5, 1.7, H-22), 3.01 (^1^H, ddd, J = 11.6, 7.1, 2.7, H-9), 2.26 (^1^H, t, J = 9.0, H-17), 2.20 (^1^H, dd, J = 13.1, 4.2, H-5), 2.02–1.50 (m, H-1, H-4, H-11, H-12, H-15, H-16, H-23, H-24), 1.06 (3H, s, H-21), 1.05 (3H, s, H-27), 1.08 (3H, s, H-26), 0.84 (3H, s, H-19), 0.76 (3H, s, H-18).

^13^C NMR (100 MHz, CDCl_3_, δ, ppm): 203.43 (C-6), 166.03 (C-8), 121.64 (C-7), 84.16 (C-14), 77.52 (C-22), 76.82 (C-20), 69.52 (C-25), 68.03 (C-2), 68.12 (C-3), 51.37 (C-5), 50.08 (C-17), 48.04 (C-13), 42.62 (C-24), 38.64 (C-10), 34.43 (C-9), 32.41 (C-4), 31.96 (C-12), 31.98 (C-15), 29.99 (C-26), 30.09 (C-27), 27.45 (C-23), 24.46 (C-19), 21.68 (C-21), 21.47 (C-16), 17.87 (C-18).

^1^H and ^13^C NMR Data of the Inclusion Complex of 20E Triacetate with β-Cyclodextrin.

The NMR spectroscopic study of the inclusion complex of 20-hydroxyecdysone triacetate with β-cyclodextrin was carried out in DMSO-d_6_, due to the good solubility of the complex and the possibility of simultaneously observing signals from both the guest molecule and the macrocyclic host (Table 1).

The ^1^H NMR spectra of the complex generally retain the characteristic set of signals of 20-hydroxyecdysone triacetate, including the resonances of the oxygenated protons H-2 (δ ≈ 4.94 ppm), H-3 (δ ≈ 5.16 ppm), and H-22 (δ ≈ 4.63 ppm), as well as the signals of the methyl groups at C-18, C-19, C-21, C-26, and C-27. At the same time, upon complex formation, minor but reproducible changes in chemical shifts (Δδ up to 0.03 ppm) are observed, indicating weak noncovalent interactions between the triacetate molecule and the cavity of β-cyclodextrin.

The signals of the acetyl methyl groups (δ ≈ 1.87–2.04 ppm) and the carbonyl carbon atoms of the ester fragments (δ ≈ 170–173 ppm) remain essentially unchanged, suggesting that the acetate substituents do not directly participate in interactions with the macrocycle.

In the spectra of β-cyclodextrin, complex formation is accompanied by diagnostic changes in the chemical shifts of the internal protons H-3 and H-5, as well as the corresponding carbon atoms, whereas the signals of the external protons (H-1, H-2, H-4, H-6) exhibit smaller changes. These spectral features are characteristic of inclusion complex formation and indicate partial insertion of the steroidal fragment of 20-hydroxyecdysone triacetate into the hydrophobic cavity of β-cyclodextrin.

Thus, the combined data from one-dimensional ^1^H and ^13^C NMR spectroscopy confirm the formation of the inclusion complex of 20-hydroxyecdysone triacetate with β-cyclodextrin in solution.

^1^H and ^13^C NMR data of the ketoxime derivative of 20-hydroxyecdysone and its inclusion complex with β-cyclodextrin

To confirm the structure of the obtained Z-oxime derivative of 20E, ^1^H and ^13^C NMR spectra were recorded at 500 MHz (^1^H) and 125 MHz (^13^C) in deuterated dimethyl sulfoxide (DMSO-d_6_). Comparison of the obtained data with literature spectra of ecdysteroid oximes made it possible to determine the oxime configuration and confirm the successful ketone to oxime transformation [18].

The complete spectral data, including chemical shifts, multiplicities, and coupling constants, are presented below.

**^1^H NMR (500 MHz, DMSO-d_6_, δ, ppm, J/Hz):** 5.66 (^1^H, d, J = 2.6, H-7), 3.78 (^1^H, br s, H-3), 3.63 (^1^H, ddd, J = 11.9, 4.3, 3.1, H-2), 3.15 (^1^H, dd, J = 10.5, 1.7, H-22), 3.04 (^1^H, ddd, J = 11.6, 7.1, 2.7, H-9), 2.28 (^1^H, t, J = 9.0, H-17), 2.18 (^1^H, dd, J = 13.1, 4.2, H-5), 2.05–1.55 (m, H-1, H-4, H-11, H-12, H-15, H-16, H-23, H-24), 1.08 (3H, s, H-21), 1.07 (3H, s, H-27), 1.09 (3H, s, H-26), 0.85 (3H, s, H-19), 0.77 (3H, s, H-18), 10.8 (^1^H, br s, N–OH).

**^13^C NMR (125 MHz, DMSO-d_6_, δ, ppm):** 154.0 (C-6), 166.0 (C-8), 122.0 (C-7), 84.2 (C-14), 77.5 (C-22), 76.8 (C-20), 69.5 (C-25), 68.0 (C-2), 68.1 (C-3), 36.0 (C-5), 50.1 (C-17), 48.0 (C-13), 42.6 (C-24), 38.6 (C-10), 34.4 (C-9), 32.4 (C-4), 31.9 (C-12), 31.9 (C-15), 30.0 (C-26), 30.1 (C-27), 27.4 (C-23), 24.5 (C-19), 21.7 (C-21), 21.5 (C-16), 17.9 (C-18).

The signal of the C-6 carbon atom (154.0 ppm) confirms the formation of the oxime group, whereas the position of the methine carbon signal C-5 (36.0 ppm) indicates the Z-configuration of the oxime, in which the H-5 proton is in a syn-position relative to the oxime hydroxyl group. In the ^1^H NMR spectrum (Supplementary Materials), a broad N–OH signal at δ 10.8 ppm is also observed, which is characteristic of oxime derivatives in DMSO-d_6_.

To investigate the ability of the obtained 20-hydroxyecdysone ketoxime to form inclusion complexes, ^1^H and ^13^C NMR spectra of its system with β-cyclodextrin were recorded. Comparative analysis of the chemical shifts of the free ketoxime and the compound in the presence of β-cyclodextrin revealed changes associated with complex formation and confirmed the formation of a host–guest-type inclusion complex (Table 2).

Based on the data presented in the table, it was established that the simultaneous presence of the 20-hydroxyecdysone ketoxime and β-cyclodextrin in solution leads to systematic but minor changes in chemical shifts in the ^1^H and ^13^C NMR spectra. The most pronounced shifts were observed for the H-3 and H-5 protons of β-cyclodextrin, located within its hydrophobic cavity, which is a characteristic feature of inclusion complex formation.

At the same time, the chemical shifts of the protons and carbons of the steroidal framework of the ketoxime change only slightly, indicating the absence of covalent transformations and the preservation of the guest molecule structure. The combined data thus indicate the formation of a host–guest-type complex stabilized by hydrophobic interactions and hydrogen bonding.

### 2.3. Computational Study of Cyclodextrin Clathrates with 20-NOH and 3Ac-20E

**Figure 2:**
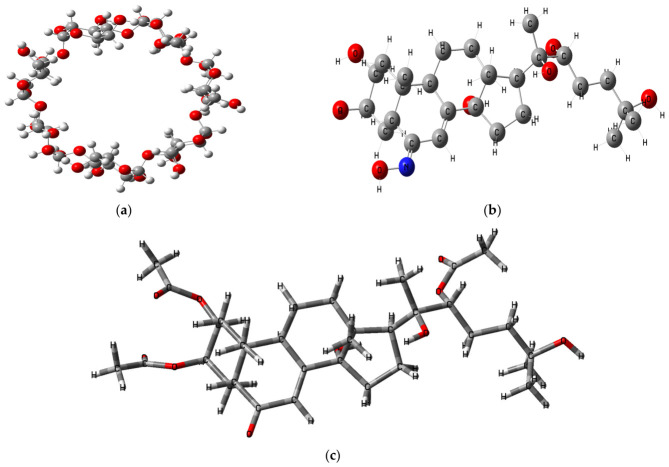
Optimal geometry of 3D molecules of *β*-CD (**a**, top view), 20-NOH·β-CD (**b**) and 3Ac-20E·β-CD (**c**) (color designation of atoms: gray—carbon, red—oxygen, blue—nitrogen, white—hydrogen).

The optimized structures of β-cyclodextrin (a), 20-NOH (b), and 3Ac-20E (c) are shown in Figure 2.

**Figure 3:**
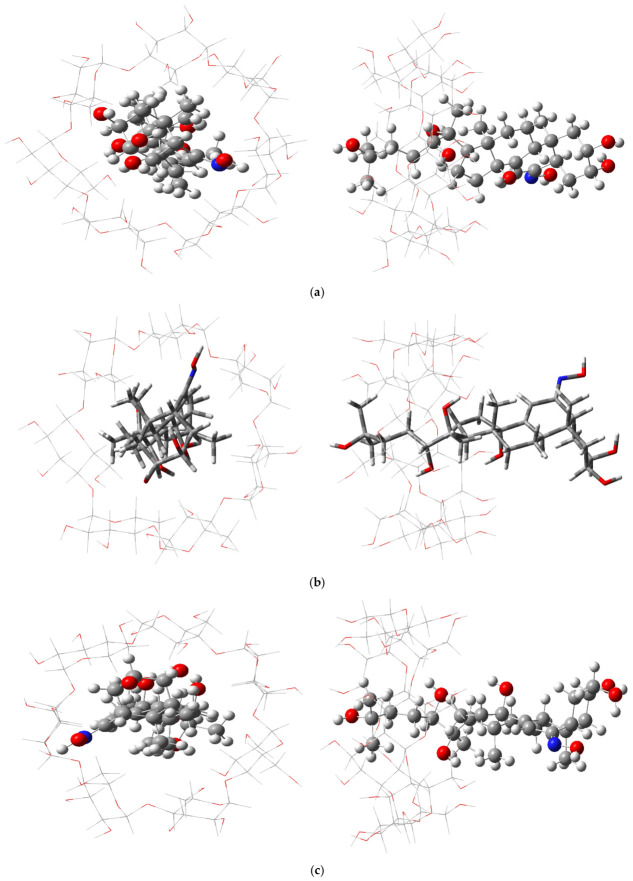

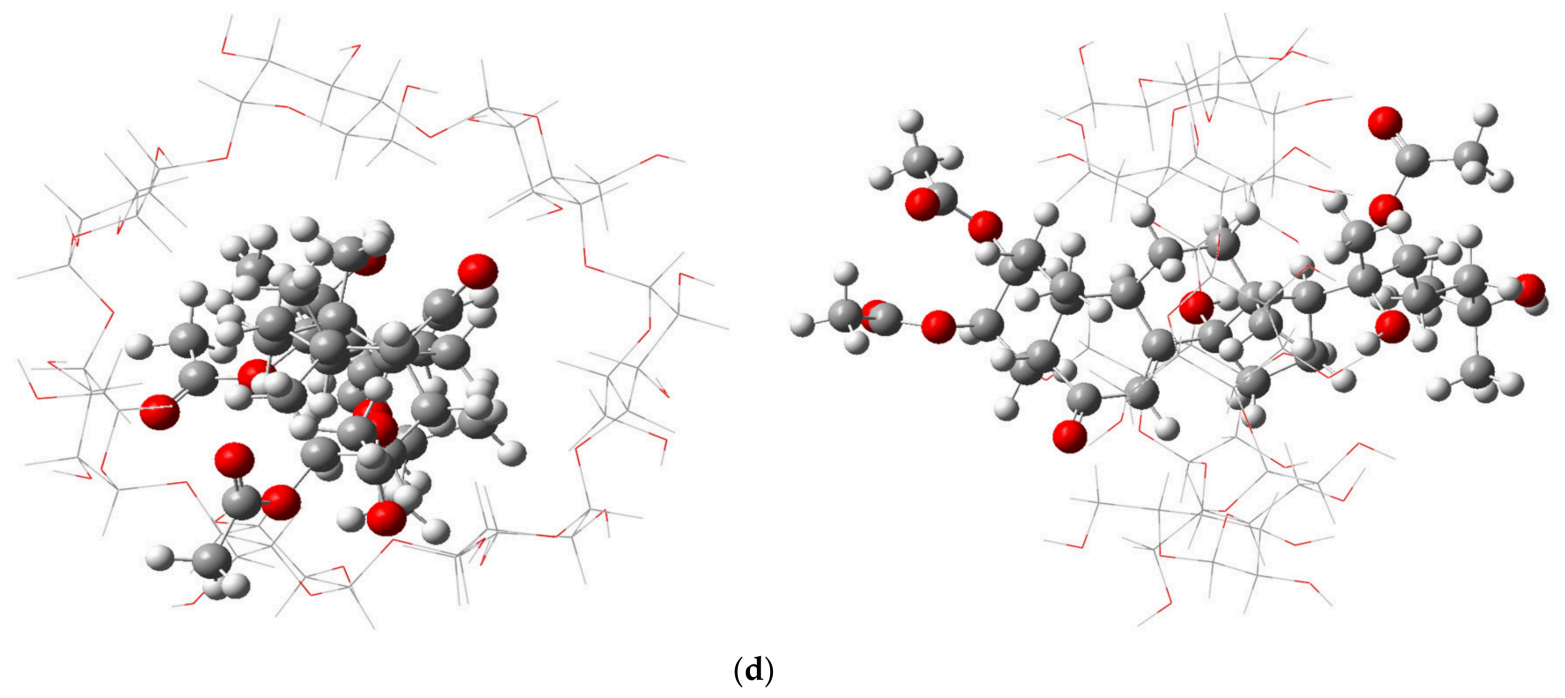
Stable configurations (top and side views) of 20-NOH inclusion complexes (ONIOM method [B3LYP/6-31G:UFF]): (**a**) “guest” on the wide side (top), (**b**) symmetrically relative to *β*-CD (central), (**c**) on the narrow side of cyclodextrin (bottom), and (**d**) 3Ac-20E “guest” symmetrically relative to *β*-CD (central).

20-NOH inclusion complexes are presented in three orientations of the guest molecule relative to the host molecule (*β*-CD). The guest molecules were viewed from the wide side of the cyclodextrin, from the narrow side, and symmetrically relative to the *β*-CD, after which the resulting systems were optimized. The most stable configurations of the three types of 20-NOH and 3Ac-20E complexes are shown in Figure 3. As can be seen from the figures, all three guest molecule incorporation options within the CD cavity are possible for the 20-NOH complex. However, optimization of the complex geometry results in nearly identical localization of the 20-NOH molecule relative to the cyclodextrin: the bulky cyclic fragment with the branched end remains outside the cavity.

The situation is different for the 3Ac-20E complex. Due to the fact that the 3Ac-20E molecule contains additional branched acetyl groups at both ends, the only possible configuration of the inclusion complex is a guest molecule positioned nearly symmetrically relative to the *β*-CD (Figure 3d).

Table 3 lists energy quantities of the host–guest complexes. The stabilization energy was calculated as the difference between the sum of the energies of the individual host and guest molecules and the energy of the inclusion complex.

As follows from Table 1, the most stable 20-NOH inclusion complex is complex Figure 3b, which has the minimum total energy. The energy difference between complexes (a) and (b) and (c) is 4 and 11 kj/mol, respectively. The stabilization energy is also highest in absolute value for complex (b), indicating a favorable symmetry of the guest molecule relative to the host molecule *β*-CD, and is negative, indicating the exothermic nature of the process of insertion of the 20-NOH molecule into the CD cavity. Interactions between the host and guest molecules are usually noncovalent. No hydrogen bonding was detected in the calculations performed. A possible cause may be repulsion between the hydrophobic internal cavity of the cyclodextrin and the hydrogen atoms at the carbon atoms of the guest molecule. The stabilization energy for the 3Ac-20E molecule complex also indicates a favorable placement of the guest molecule.

For the chemical and optical properties of a substance, two levels are most important: the highest (in energy) occupied molecular orbital (HOMO) and the lowest unoccupied molecular orbital (LUMO). Their energies are also given in Table 3.

For all systems considered, other molecular descriptors were also determined, such as zero-point energy (*E*_0_), dipole moment (*μ*), and thermodynamic parameters—entropy (*S*) and molar heat capacity (*C_v_*) (Table 4).

The nature of the change in the given quantities from complex (a) to complex (c) is non-monotonic, except for the consistently decreasing value of heat capacity. Similar characteristics for complex (d) have comparable values.

### 2.4. Biological Activity

After identifying the most efficient method for obtaining encapsulated forms, we also investigated the antioxidant, hepatoprotective, antimicrobial, and antifungal properties of 20E and its complexes obtained under the selected conditions. This is due to the fact that antioxidant bioactivity plays a key role in the manifestation of biological effects and may significantly determine functional properties, including hepatoprotective potential, as well as the ability to inhibit the growth of microorganisms and fungi.

#### 2.4.1. FRAP Antioxidant Assay

The reducing antioxidant activity of the studied compounds was determined using the FRAP assay, based on the ability to reduce Fe^3+^ ions to Fe^2+^. The results were expressed as ascorbic acid equivalents (AAE/mL), allowing for an evaluation of the reducing potential of the tested compounds.

The obtained results revealed differences in reducing capacity between the parent compound and its β-cyclodextrin complexes. For 20E, FRAP values remained relatively low across the entire investigated concentration range. At a concentration of 0.05 mg/mL, the value was 0.451 ± 0.037 AAE/mL, and at 0.20 mg/mL it was 0.460 ± 0.021 AAE/mL. With further increases in concentration, a decrease in reducing activity was observed, reaching 0.065 ± 0.004 AAE/mL at 0.25 mg/mL and 0.054 ± 0.004 AAE/mL at 0.75 mg/mL, indicating a relatively weak reducing capacity of the parent compound. This counterintuitive decrease likely reflects the limited aqueous solubility of 20E, which causes aggregation or precipitation at higher concentrations, reducing the effective concentration of reducing molecules in the assay system. Similar concentration-dependent non-linearity has been reported for poorly soluble polyphenols and steroids in aqueous-based FRAP assays, where solubility limits the available reducing equivalents at higher nominal concentrations [19,20].

In contrast, 20-NOH·β-CD demonstrated significantly higher reducing activity. The FRAP values increased from 1.304 ± 0.525 AAE/mL (0.05 mg/mL) to 3.452 ± 0.520 AAE/mL (0.75 mg/mL), indicating a pronounced ability of the complex to reduce iron ions. At 1.0 mg/mL, the value was 2.829 ± 0.124 AAE/mL.

The highest reducing activity was observed for and 3Ac-20E·β-CD. At a concentration of 0.25 mg/mL, the value reached 3.679 ± 0.182 AAE/mL, and with further increases in concentration, it remained at a high level, reaching 3.810 ± 0.279 AAE/mL at 1.0 mg/mL (Table 5).

Analysis of the concentration dependence showed that the EC_50_ values were approximately 0.18 mg/mL for 20E, 0.17 mg/mL for 20-NOH, and 0.16 mg/mL for and 3Ac-20E·β-CD, indicating higher antioxidant activity of the β-cyclodextrin complexes compared to the parent compound.

#### 2.4.2. DPPH Radical Scavenging Activity

The radical scavenging activity of 20-hydroxyecdysone and its β-cyclodextrin complexes was evaluated using the 2,2-diphenyl-1-picrylhydrazyl (DPPH) assay. The obtained results demonstrated a concentration-dependent increase in the antiradical activity of the studied compounds.

The parent compound, 20E, exhibited relatively moderate DPPH radical scavenging activity. At low concentrations (0.05–0.20 mg/mL), the inhibition of DPPH radicals remained minimal and did not exceed 2%. A significant increase in activity was observed at higher concentrations, particularly from 0.25 mg/mL onward. At concentrations of 0.25, 0.50, 0.75, and 1.00 mg/mL, the inhibition values reached 66.0%, 70.3%, 73.8%, and 79.1%, respectively. According to the concentration–response curve, the IC_50_ value for 20-hydroxyecdysone was 0.24 mg/mL, indicating moderate antiradical activity.

In contrast, the β-cyclodextrin inclusion complexes of 20E derivatives demonstrated significantly higher radical scavenging activity. The 20-NOH·β-CD and 3Ac-20E·β-CD complexes exhibited pronounced inhibition of DPPH radicals even at the lowest concentration tested (0.05 mg/mL). The inhibition values for the 20-NOH complex ranged from approximately 91.6% to 95.9%, while the 3Ac-20E·β-CD complex showed inhibition values from 88.8% to 95.9% across the entire concentration range studied. In both cases, the IC_50_ values were estimated to be below 0.05 mg/mL, indicating a substantially enhanced radical scavenging capacity compared to the parent compound 20E and the reference standard BHA (Table 5).

Overall, the obtained results demonstrate that complexation of 20-hydroxyecdysone derivatives with β-cyclodextrin significantly enhances their antiradical activity. Among the studied samples, the β-cyclodextrin complexes exhibited the highest DPPH radical scavenging capacity, highlighting the potential of cyclodextrin-based complexes to improve the antioxidant properties of biologically active compounds.

The obtained results indicate that 20-NOH·β-CD and 3Ac-20E·β-CD exhibit more pronounced antioxidant activity compared to the parent compound 20E. It should be noted that ecdysteroids do not contain phenolic hydroxyl groups, which are typically responsible for the high antioxidant activity of natural compounds. Nevertheless, their antioxidant potential has been confirmed in a number of studies [21,22,23,24]. In the present study, the higher EC_50_ and IC_50_ values observed for 20E are likely due to its low solubility, which limits the availability of molecules for interaction with DPPH radicals and Fe^3+^ ions. In contrast, the formation of inclusion complexes with β-cyclodextrin leads to a significant increase in solubility and dispersibility of the derivatives, thereby enhancing their antioxidant activity [25,26]. This is confirmed by the substantially lower EC_50_ and IC_50_ values for the ketoxime and acetate complexes, indicating greater efficiency in free radical scavenging and in the reduction of Fe^3+^ to Fe^2+^. Thus, complexation with β-cyclodextrin can be considered an effective strategy for enhancing the antioxidant potential of ecdysteroids.

#### 2.4.3. Hepatoprotective Activity

During the observation period, the general condition of the animals remained relatively stable. Compared with the other groups, the animals in the control group exhibited general signs of intoxication: lethargy, reduced activity, loss of appetite and weight loss. CCl_4_ intoxication was accompanied by marked changes in the animals’ body weight and liver weight. In the control group, a significant decrease in body weight was observed (from 279.8 ± 18.0 to 190.8 ± 25.0 g), along with an increase in liver weight (10.3 ± 2.5 g) and liver index (5.4%), indicating the development of toxic liver damage. In the intact group, these parameters remained stable (liver index—3.0%).

Against the background of administration of the reference drug Carsil, normalization of these parameters was observed: body weight increased (from 168.5 ± 36.7 to 177.5 ± 44.2 g), liver weight decreased to 5.5 ± 1.3 g, and the liver index was 3.1%, approaching the values of the intact group.

In the experimental groups receiving the studied compositions, a positive trend was also observed compared to the control. Body weight either increased or showed only a slight decrease (for example, at doses of 25–75 mg/kg: 185.7 ± 60.9 → 196.7 ± 42.2 g; 223.3 ± 11.4 → 217.3 ± 14.7 g). Liver weight ranged from 5.5 to 8.8 g, and the liver index decreased to 2.3–3.8%, indicating a reduction in the severity of pathological changes.

To comprehensively assess liver condition and confirm morphometric changes, biochemical analyses of blood serum and liver homogenates were performed, along with ultrasound and histological examinations.

Ultrasound examination of the liver showed that animals in the intact group retained a normal echostructure without signs of pathological changes. In the control group exposed to CCl_4_, pronounced structural alterations were detected, including parenchymal heterogeneity, signs of fatty infiltration, and hypoechoic inclusions, indicating the development of toxic liver injury [27]. In the experimental groups, the ultrasound pattern of the liver was close to normal: the organ structure remained homogeneous, with no evidence of steatosis or pathological inclusions.

The functional state of the liver was assessed using biochemical parameters. The activities of alanine aminotransferase (ALT) and aspartate aminotransferase (AST) were determined as markers of hepatocellular damage [28], along with the concentration of malondialdehyde (MDA) in liver homogenates, which reflects the level of lipid peroxidation [29].

As shown in Table 6, the administration of CCl_4_ led to the development of pronounced toxic liver injury. In the control group, ALT activity increased to 153.6 ± 0.3 U/L, whereas in the intact group this parameter was 135.6 ± 0.5 U/L. A similar trend was observed for AST activity, which increased from 71.9 ± 0.1 U/L in the intact group to 79.9 ± 0.2 U/L in the control group. The elevation of transaminase activity indicates disruption of hepatocyte membrane integrity and the release of intracellular enzymes into the bloodstream. At the same time, the De Ritis ratio remained below unity (0.52), suggesting a predominantly cytoplasmic nature of liver cell damage [30]. In addition, an increase in MDA levels to 0.60 ± 0.01 µmol/L was observed, indicating activation of lipid peroxidation processes and the development of oxidative stress.

In the group of animals treated with the reference drug Carsil, a decrease in transaminase activity was observed compared to the toxic control group. ALT and AST values were 121.4 ± 0.4 and 73.7 ± 0.3 U/L, respectively, while MDA levels decreased to 0.57 ± 0.01 µmol/L, confirming the hepatoprotective and antioxidant activity of the drug.

Analysis of the experimental groups receiving the studied compounds revealed a dose-dependent but heterogeneous pattern of effects. The administration of 20-hydroxyecdysone was accompanied by a decrease in ALT activity with increasing dose. Specifically, at doses of 25, 50, and 75 mg/kg, ALT values were 132.5 ± 0.5, 114.7 ± 0.4, and 106.7 ± 0.2 U/L, respectively, indicating a reduction in the degree of hepatocellular damage. AST values at these doses were 60.8 ± 0.2, 73.8 ± 0.2, and 76.8 ± 0.3 U/L. However, MDA levels in these groups were significantly higher compared with the toxic control group (1.07–1.82 µmol/L), which may indicate insufficient suppression of lipid peroxidation processes and limited antioxidant efficacy of the compound under the conditions of this model [31].

The most pronounced changes in the studied parameters were observed following the administration of the inclusion complex of the ketoxime derivative of 20-hydroxyecdysone with β-cyclodextrin (20-NOH·β-CD). At a dose of 25 mg/kg, ALT activity decreased to 97.7 ± 0.1 U/L, while the MDA level was 0.07 ± 0.01 µmol/L, which is significantly lower compared with the toxic control group. Such a marked reduction in MDA concentration indicates effective suppression of lipid peroxidation processes and protection of hepatocyte membranes [32]. At a higher dose of 50 mg/kg, a slight increase in ALT activity (116.7 ± 0.2 U/L) and MDA levels (0.92 ± 0.01 µmol/L) was observed, whereas at a dose of 75 mg/kg, a significant increase in ALT (179.7 ± 0.4 U/L) and MDA (3.24 ± 0.01 µmol/L) was detected. These findings indicate the absence of a strict linear dose–response relationship and may suggest the presence of an optimal therapeutic dose for this compound, as well as possible dose-dependent adverse or pro-oxidant effects at higher concentrations.

For the inclusion complex of the acetate derivative of 20-hydroxyecdysone with β-cyclodextrin (3Ac-20E·β-CD), a decrease in ALT activity with increasing dose was also observed. ALT values were 224.3 ± 0.4, 126.9 ± 0.1, and 116.9 ± 1.6 U/L at doses of 25, 50, and 75 mg/kg, respectively. Despite the reduction in transaminase activity, MDA levels remained relatively high (0.82–1.93 µmol/L), indicating less pronounced suppression of lipid peroxidation processes.

These results are consistent with the in vitro antioxidant activity data obtained in the DPPH assay. The parent compound, 20-hydroxyecdysone, exhibited moderate free radical scavenging capacity, whereas the β-cyclodextrin complexes of its derivatives demonstrated significantly higher activity, achieving 88.8–95.9% inhibition of radicals even at low concentrations (IC_50_ < 0.05 mg/mL). The enhanced antiradical activity of these complexes contributes to more effective suppression of free radical processes induced by CCl_4_ [33].

The ultrasound findings are consistent with the results of the biochemical analysis, where the control group showed elevated levels of transaminases (ALT, AST), indicating damage to hepatocyte membranes and the release of enzymes into the bloodstream. In contrast, the hepatoprotective activity of the studied samples was accompanied by normalization of biochemical parameters—decreased ALT and AST levels, the restoration of antioxidant status, the stabilization of cellular membranes, and the prevention of structural liver damage.

Thus, the conducted studies demonstrated that among the investigated compounds, the inclusion complex of the ketoxime derivative of 20-hydroxyecdysone with β-cyclodextrin demonstrated the most notable hepatoprotective and antioxidant activity, especially at a dose of 25 mg/kg, where the greatest reduction in transaminase activity and the lowest MDA levels were observed. At the same time, the efficacy of the compounds depended on dose, and higher concentrations in some cases were associated with reduced protective effects and possible adverse outcomes.

#### 2.4.4. Histology

**Figure 4:**
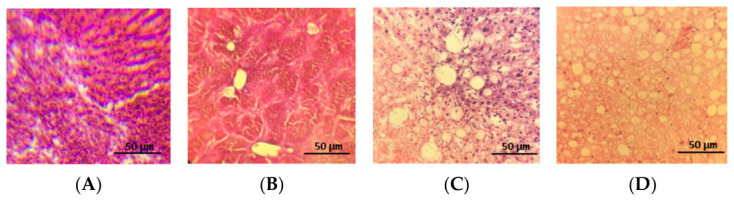
Histological sections of rat liver from experimental groups (hematoxylin and eosin staining): (**A**) intact group; (**B**) control group (CCl_4_ intoxication); (**C**) experimental group (20E, 25 mg/kg); (**D**) experimental group (20-NOH·β-CD, 25 mg/kg). Magnification: ×800. Scale bar = 50 μm.

The histological findings of the liver in different experimental groups are presented in Figure 4. The results showed that animals in the intact group retained a normal liver architecture: hepatic lobules, radially arranged hepatic cords, central veins, and sinusoidal capillaries were clearly visualized. Hepatocytes exhibited a typical polygonal shape with well-defined nuclei, and no signs of dystrophic changes were observed.

In the control group subjected to CCl_4_ intoxication, pronounced pathological alterations were detected, including disruption of the hepatic cord architecture, sinusoidal dilation, congestion of central veins, and marked fatty degeneration of hepatocytes, characterized by cytoplasmic vacuolization and peripheral displacement of nuclei. Inflammatory infiltration and disorganization of the hepatic parenchyma were also noted [34].

In the experimental groups treated with the studied compounds 20E and 20-NOH·β-CD at a dose of 25 mg/kg, partial restoration of liver morphology was observed. The hepatic architecture was largely preserved, the degree of fatty degeneration was reduced, and vascular disturbances and inflammatory infiltration were less pronounced. Hepatocytes appeared more organized, with fewer vacuoles and reduced signs of cellular damage [35].

The observed morphological changes are consistent with the biochemical findings, reflecting normalization of transaminase activity and MDA levels, indicating reduced hepatocellular damage and oxidative stress. These results are also in agreement with the ultrasound data, suggesting restoration of the liver structure. Overall, the studied compounds demonstrated signs of hepatoprotective activity, with 20-NOH·β-CD at a dose of 25 mg/kg showing comparatively greater protective effects, contributing to structural preservation of liver tissue and a reduction in biochemical markers of injury.

A comprehensive assessment of the liver showed that ultrasound changes in the control group corresponded to histological signs of hepatocellular dystrophy and increased biochemical markers of damage (ALT, MDA). In turn, the use of the studied compounds, especially 20-hydroxyecdysone with β-cyclodextrin (25 mg/kg), led to the normalization of liver size, alignment of contours, improvement in echostructure, restoration of morphological integrity of tissue and reduction in biochemical parameters, suggesting a hepatoprotective effect. This effect can be explained by its chemical structure of the compound: polar hydroxyl groups provide an antioxidant effect, reducing LPO and protecting hepatocyte membranes, and the complex with β-cyclodextrin increases the solubility and bioavailability of the compound, contributing to more effective penetration into liver cells. These properties directly affect the macro- and microstructure of the liver, normalization of size, improvement in echostructure and reduction in biochemical markers of damage.

#### 2.4.5. Antibacterial and Antifungal Activity

The antimicrobial and antifungal activity of the studied compounds was evaluated using the agar diffusion method and the broth microdilution method with determination of minimum inhibitory concentrations (MIC). The use of these two approaches allowed for the simultaneous assessment of the ability of the compounds to diffuse in a solid medium and their activity in a liquid system. The obtained results are presented in Table 7.

According to the agar well diffusion assay, the studied compounds exhibited varying degrees of activity against the tested microorganisms. The highest antibacterial activity was observed for 20E, which demonstrated pronounced inhibition zones against *S. aureus* and *E. coli*, as well as moderate activity against *K. pneumoniae* and *P. aeruginosa*. In contrast to the parent compound, 20-NOH·β-CD exhibited moderate activity against all tested strains. The 3Ac-20E·β-CD compound showed almost no antibacterial activity against most of the tested strains (*E. coli*, *S. aureus*, *K. pneumoniae*). Slight activity was observed only against *P. aeruginosa*, with an inhibition zone diameter of approximately 12.7 ± 0.6 mm.

**Figure 5:**
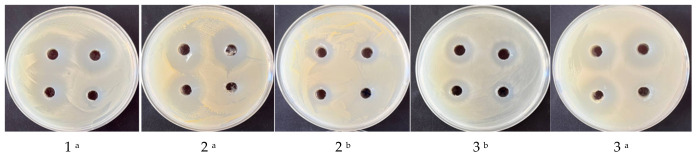
Antimicrobial activity of 20E and its derivatives against selected microorganisms evaluated by using agar well diffusion method: 1—*E. coli*, 2—*S. aureus*, and 3—*K. pneumoniae*. Here, 20E ^a^, 20-NOH·β-CD ^b^.

A different pattern was observed for the fungal strain *Candida albicans*. According to the agar diffusion results, 20E did not exhibit inhibitory activity, whereas 20-NOH·β-CD showed moderate antifungal activity and 3Ac-20E·β-CD exhibited weak activity (Figure 5).

Quantitative evaluation using the MIC method showed that for all studied compounds, MIC values against bacterial strains exceeded 10 mg/mL, indicating the absence of pronounced antibacterial activity within the tested concentration range. Against *Candida albicans*, the most notable effect was observed for 20E, with an MIC value of approximately 10 mg/mL, whereas for 20-NOH·β-CD and 3Ac-20E·β-CD the MIC values exceeded this level.

The obtained results demonstrate differences between the methods used to assess antimicrobial activity. Agar diffusion is strongly influenced by compound solubility, molecular size, and the ability to diffuse through a solid medium, whereas the MIC method more accurately reflects the ability of a substance to completely inhibit microbial growth in a liquid phase [36,37]. In this case, this distinction is particularly important, as structural modification of 20E affects not only its biological properties but also its physicochemical parameters.

Structurally, 20E is a more polar compound due to the presence of hydroxyl groups, whereas acetylation in 3Ac-20E·β-CD increases molecular hydrophobicity and may reduce its effective bioavailability in a liquid system. The introduction of a ketoxime function in 20-NOH·β-CD, in turn, alters the electronic structure and spatial configuration of the steroid nucleus. These modifications can differentially influence diffusion in agar and interactions with microbial cell membranes. This explains why the modified forms exhibited activity against *C. albicans* in the agar diffusion assay, while the parent compound 20E proved more effective in the MIC evaluation.

Ecdysteroids are generally not classified as strong antimicrobial agents and typically exhibit weak to moderate activity depending on their chemical structure [38,39,40,41]. In study [38], it was shown that semisynthetic oxime derivatives of 20E exhibit pronounced antifungal activity against *Cryptococcus neoformans* (MIC = 1–2 mg/mL), whereas the parent compound was less active. This supports the notion that chemical modification of ecdysteroids can either enhance or reduce activity depending on the test system and the type of microorganism.

Overall, the obtained results indicate that chemical modification of 20-hydroxyecdysone leads to multidirectional changes in its biological properties. Importantly, the MIC values exceeding 10 mg/mL for all compounds against bacterial strains indicate negligible antibacterial activity in liquid media. While β-cyclodextrin-based complexes of modified derivatives show enhanced antioxidant activity, this advantage is not retained for antimicrobial effects, particularly in liquid media. Therefore, the impact of structural modification on the bioactivity of ecdysteroids should be considered a multiparametric process dependent on the nature of the test system and the underlying mechanism of action.

## 3. Materials and Methods

### 3.1. Reagents, Chemicals and Standards

All reagents and solvents were of analytical or higher grade and were used without further purification unless otherwise stated. β-Cyclodextrin (β-CD, purity ≥ 98%, Fluka, Adachi, Japan), acetic anhydride (Ac_2_O, purity ≥ 99%), anhydrous pyridine (purity ≥ 99.8%), hydroxylamine hydrochloride (NH_2_OH·HCl, purity ≥ 99%), sodium bicarbonate (NaHCO_3_, purity ≥ 99.7%), and carbon tetrachloride (CCl_4_, purity ≥ 99.5%) were obtained from Sigma-Aldrich (St. Louis, MO, USA). Solvents (petroleum ether, ethyl acetate, chloroform, absolute ethanol, and isobutanol) were purchased from local suppliers. Deuterated solvents for NMR spectroscopy (CDCl_3_ and DMSO-d_6_, 99.8 atom %D) were purchased from Sigma-Aldrich. All aqueous solutions were prepared using deionized water (resistivity ≥ 18.2 MΩ·cm) from a Milli-Q purification system (Merck Millipore, Burlington, MA, USA).

2,2-Diphenyl-1-picrylhydrazyl (DPPH, purity ≥ 96%), potassium ferricyanide (K_3_[Fe(CN)_6_], purity ≥ 99%), iron(III) chloride (FeCl_3_, purity ≥ 99%), trichloroacetic acid (TCA, purity ≥ 99%), and thiobarbituric acid (TBA, purity ≥ 98%) were obtained from Sigma-Aldrich. A phosphate buffer (0.2 M, pH 6.6) was prepared in the laboratory from sodium dihydrogen phosphate and disodium hydrogen phosphate (both purity ≥ 99%, Sigma-Aldrich). Nutrient broth and Mueller–Hinton agar were obtained from HiMedia Laboratories (Mumbai, India). Silymarin (Carsil) was used in the form of the commercial preparation Carsil^®^ (70 mg film-coated tablets, Sopharma AD, Sofia, Bulgaria) as a reference hepatoprotective drug. Butylated hydroxyanisole (BHA, purity ≥ 98%, Sigma Aldrich) and L-ascorbic acid (purity ≥ 99%, Sigma-Aldrich).

### 3.2. Isolation of 20-Hydroxyecdysone

The aerial parts of *Silene wolgensis* Hornem. Bess. ex Spreng. were collected in the Bukhar-Zhyrau district of the Karaganda region, Republic of Kazakhstan, during the flowering stage in 2025. The plant species was identified by Dr. Sci. (Biol.), Professor A.N. Kupriyanov, Director of the Kuzbass Botanical Garden, Institute of Human Ecology SB RAS (Kemerovo), in collaboration with researchers from the Laboratory of Botany and Biotechnology of JSC “Phytochemistry”. Voucher specimens are deposited in the Herbarium Fund of JSC “Phytochemistry”.

Air-dried plant material (2.0 kg), ground to a particle size of 2–3 mm, was extracted with 96.2% ethanol at a fourfold solvent-to-material ratio. The extraction was performed three times. The combined ethanolic extracts (total dry residue of approximately 115 g) were treated with hot ethanol to remove chlorophyll, followed by successive washing with petroleum ether and extraction with isobutanol. As a result, a syrupy mass (55 g) was obtained, which was subjected to separation by high-performance liquid chromatography (HPLC) using chloroform–alcohol eluent systems in ratios of 30:1 (fractions 1–22) and 5:1 (fractions 23–50). Fractions 1–22 contained the minor phytoecdysteroid 2-deoxyecdysone, which was isolated by recrystallization from ethyl acetate. From fractions 23 to 50, 3.0 g of 20-hydroxyecdysone was isolated with a purity of 98.2%.

20-Hydroxyecdysone: mp 236–238 °C (ethyl acetate–methanol); [α]D^20^ 66.0 (c 1.0, MeOH). IR (KBr), ν, cm^−1^: 3320 (OH), 2950 (–CH_2_–), 1650 (C=O, conjugated), 1450, 1200, 1080, 880. UV (EtOH), λ_max, nm: 244 (log ε 4.10).

### 3.3. Synthesis of 2,3,22-Triacetyl-20-Hydroxyecdysone and Its Inclusion Complex with β-Cyclodextrin

2,3,22-Triacetate of 20-hydroxyecdysone (3Ac-20E) was obtained by acetylation of 20-hydroxyecdysone with acetic anhydride (Ac_2_O) in anhydrous pyridine at a molar ratio of starting compound to Ac_2_O of 1:100. As a result of the reaction, ecdysterone derivatives were obtained: 2,3,22-triacetate in 65% yield and 2,3,22,25-tetraacetate in 29% yield, respectively, in accordance with the previously reported procedure [42].

The inclusion complex of 3Ac-20E with β-cyclodextrin was prepared by interaction of equimolar amounts of the components. A solution of 3Ac-20E (0.050 g, 0.083 mmol) in 3 mL of absolute ethanol was mixed with a solution of β-cyclodextrin (0.094 g, 0.083 mmol) in 4 mL of distilled water. The reaction mixture was stirred on a magnetic stirrer at 50 °C for 8 h. The resulting precipitate was filtered, washed with ethanol, and dried in a vacuum drying oven at 40 °C. The inclusion complex was obtained as a white powder.

IR (KBr), ν, cm^−1^: 579, 609, 707, 757, 858, 947, 1029, 1081, 1157, 1216, 1254. UV (λ_max): 245 nm. mp: 267 °C (decomp.). The formation of the inclusion complex was confirmed by one- and two-dimensional ^1^H–^13^C NMR spectroscopy.

### 3.4. Synthesis of Ketoxime Derivative of 20-Hydroxyecdysone and Its Inclusion Complex

The ketoxime derivative of 20-hydroxyecdysone (20-NOH) was obtained by oximation of the carbonyl group of the parent compound with hydroxylamine. For this purpose, 20-hydroxyecdysone (0.050 g, 0.083 mmol) was dissolved in 3 mL of absolute ethanol. Hydroxylamine hydrochloride was pretreated with an equimolar amount of sodium bicarbonate in a minimal volume of distilled water to generate free hydroxylamine in situ. The resulting hydroxylamine solution was added to the solution of 20-hydroxyecdysone under constant stirring. The reaction mixture was stirred for 72 h at 50 °C according to a previously reported procedure [43]. The resulting crystalline product was isolated and dried under vacuum.

For the preparation of the inclusion complex, 0.05 g (0.00011 mmol) of the ketoxime derivative of 20-hydroxyecdysone was dissolved in 5 mL of absolute ethanol. A solution of β-cyclodextrin (0.114 g, 0.00011 mmol), previously dissolved in water, was added to the mixture. The reaction mixture was stirred on a magnetic stirrer at 40–50 °C for 8 h. The resulting crystalline precipitate was filtered, washed with ethanol, and dried under vacuum.

### 3.5. NMR Spectroscopic Study of Structure and Inclusion Complex Formation

NMR analysis was performed on a Jeol JNM-ECA 500 spectrometer (JEOL, Tokyo, Japan). The spectra of 20E, 3Ac-20E, and 3Ac-20E·β-CD were recorded at room temperature at operating frequencies of 400 MHz for ^1^H and 100 MHz for ^13^C in DMSO-d_6_ (for complexes) or CDCl_3_ (for 20E). The spectra of 20-NOH and 20-NOH·β-CD were recorded at operating frequencies of 500 MHz for ^1^H and 125 MHz for ^13^C in DMSO-d_6_. Chemical shifts (δ) are reported in ppm relative to residual solvent signals.

^1^H and ^13^C NMR spectra of 20-hydroxyecdysone (20E) were recorded in deuterated chloroform (CDCl_3_), selected due to the good solubility of the compound and the ability to obtain high-resolution spectra. For the inclusion complex of 20-hydroxyecdysone triacetate with β-cyclodextrin, spectra were recorded in DMSO-d_6_, which provided sufficient solubility of the complex and allowed for simultaneous observation of signals from both the guest molecule and the host.

To confirm the structure of the synthesized Z-oxime derivative of 20E, ^1^H and ^13^C NMR spectra were recorded in deuterated dimethyl sulfoxide (DMSO-d_6_). Comparison of the obtained spectra with literature data for ecdysteroid oximes was used to establish the configuration of the oxime group and to confirm the conversion of the ketone into the oxime.

### 3.6. Computational Study of Cyclodextrin Clathrates with 20-NOH and 3Ac-20E

To study the stability and properties of inclusion complexes (clathrate), the initial geometries of cyclodextrin (*β*-CD), the ketoxime derivative of 20-hydroxyecdysone (20-NOH), and 2,3,22-triacetate of 20-hydroxyecdysone (3Ac-20E) were fully optimized using the semiempirical quantum chemical method DFT/B3LYP/6-31G [44]. Optimization was performed without imposing symmetry conditions. The resulting structures were identified as minima on the potential energy surface. However, inclusion complexes are bulk systems, so the hybrid ONIOM (B3LYP/6-31G:UFF) method, which combines quantum and molecular mechanics methods, was used to characterize them. All calculations were performed using Gaussian 16W [44]. Figures were prepared using GaussView 6.0 [45].

### 3.7. FRAP Antioxidant Assay

The ferric-reducing antioxidant power (FRAP) of the tested samples was determined based on their ability to reduce Fe^3+^ ions to Fe^2+^ using the potassium ferricyanide reduction method [46]. Briefly, 1 mL of the sample solution at concentrations of 0.05, 0.10, 0.15, 0.20, 0.25, 0.50, 0.75, and 1.0 mg/mL was mixed with 2.5 mL of phosphate buffer (0.2 M, pH 6.6) and 2.5 mL of 1% potassium hexacyanoferrate (III) K_3_[Fe(CN)_6_]. The reaction mixture was incubated at 50 °C for 25 min. After incubation, the reaction was terminated by adding 2.5 mL of 10% trichloroacetic acid, followed by centrifugation at 3000 rpm for 3 min. Then, 2.5 mL of the supernatant was mixed with 2.5 mL of distilled water and 0.5 mL of 0.1% FeCl_3_ solution. The resulting mixture was allowed to stand for 10 min at room temperature, after which the absorbance was measured at 700 nm using a UV–Vis spectrophotometer (Agilent Cary 60, Agilent, Santa Clara, CA, USA). Ascorbic acid was used as a standard.

The results are presented as mean absorbance values ± standard deviation (SD) obtained from three independent measurements and expressed as EC_50_ values (μg/mL), defined as the concentration of the tested compound required to achieve 50% inhibition of oxidative activity.

### 3.8. DPPH Radical Scavenging Activity

The radical scavenging activity of the tested compounds was evaluated based on their ability to neutralize the stable free radical 2,2-diphenyl-1-picrylhydrazyl (DPPH) [47]. The samples were dissolved in ethanol to obtain solutions at concentrations of 0.05, 0.10, 0.15, 0.20, 0.25, 0.50, 0.75, and 1.0 mg/mL. An aliquot of 0.1 mL of each sample solution was added to 3.0 mL of a freshly prepared 0.06 mM ethanolic DPPH solution.The mixtures were thoroughly mixed and incubated in the dark at room temperature for 30 min. After incubation, the absorbance was measured at 517 nm against a blank solution (DPPH solution without sample) using a UV–Vis spectrophotometer (Agilent Cary 60, USA). The percentage of DPPH radical inhibition was calculated using the following equation:

ARA (%) = [(A_0_ − A_t_)/A_0_] × 100 (1)

where A_0_ is the absorbance of the control and A_t_ is the absorbance of the sample.

The antiradical activity was expressed as IC_50_ values (μg/mL), defined as the concentration of the extract required to inhibit 50% of the DPPH radical activity. Butylated hydroxyanisole (BHA) was used as a positive control.

### 3.9. Hepatoprotective Activity

All animal procedures were approved by the Local Ethics Committee of Astana Medical University (Protocol No. 9, dated 24 June 2025) and conducted in accordance with international guidelines. All efforts were made to minimize animal suffering and to reduce the number of animals used.

In vivo experiments were performed on outbred white rats (180–300 g, *n* = 48), maintained under standard vivarium conditions (temperature 22 ± 2 °C, relative humidity 50–60%, and a 12 h light/dark cycle) with free access to standard diet and water. The animals were randomly divided into 12 experimental groups (*n* = 4 per group):

Group 1—intact control;

Group 2—toxic control (CCl_4_-induced intoxication);

Group 3—reference group treated with silymarin (Carsil), administered at a dose of 100 mg/kg/day, dissolved in an appropriate solvent depending on the solubility of the test compound.

Experimental groups received the following compounds: 20-hydroxyecdysone (Groups 4–6, 20E), the inclusion complex of the ketoxime derivative of 20-hydroxyecdysone with β-cyclodextrin (Groups 7–9, 20-NOH), and the inclusion complex of the acetate derivative of 20-hydroxyecdysone with β-cyclodextrin (Groups 10–12, 3Ac-20E). The compounds were dissolved in 5% dimethyl sulfoxide (DMSO), which was used uniformly across all experimental groups, and administered at doses of 25–75 mg/kg/day. DMSO at low concentrations is widely used as a vehicle in in vivo studies and is generally reported to have minimal biological effects when applied consistently across experimental groups [48].

All test compounds and the reference drug were administered orally using a gastric gavage once daily for 7 consecutive days.

To induce toxic liver injury, all animals except those in the intact group received intraperitoneal injections of a 50% oily solution of CCl_4_ in sunflower oil [49]. The administered dose was 0.2 mL per 200 g of body weight, corresponding to 0.1 mL of pure CCl_4_ (≈0.5 mL/kg or ≈0.795 g/kg). Injections were performed on days 2, 4, and 6. Hepatoprotective activity was evaluated on day 7.

Liver visualization was performed using a non-invasive ultrasound examination of the liver parenchyma with a linear probe on a portable ultrasound system (Acclarix AX3, Edan). This approach allowed for an in vivo assessment of tissue structure and echogenicity. Ultrasonographic evaluation was conducted according to standard protocols reflecting morphological changes associated with toxic liver injury [50,51].

At the end of the experiment, animals were euthanized in a desiccator using chloroform. Blood samples were collected by cardiac puncture, followed by liver excision for subsequent biochemical and histological analyses. Blood samples were centrifuged at 3000 rpm for 20 min to obtain serum, which was stored at −20 °C until biochemical analysis. A portion of liver tissue was used for the preparation of homogenates for malondialdehyde (MDA) determination and was processed immediately after collection without long-term storage.

Alanine aminotransferase (ALT) and aspartate aminotransferase (AST) activities were determined using commercial kits (“Vital”) according to the Reitman–Frankel method, which is widely used in studies of hepatoprotective activity [52]. The concentration of MDA in liver homogenates, as an indicator of lipid peroxidation (LPO), was measured by differential spectrophotometry (Agilent Cary 60, USA) based on its reaction with thiobarbituric acid [53].

Throughout the experiment, animal behavior, body weight, and liver weight were monitored and recorded.

### 3.10. Histology

The liver of white laboratory rats was used as the material for histological examination. Histological sample preparation was performed in accordance with standard histotechnical procedures [54]. Animal dissection was carried out in compliance with principles of biological ethics and humane treatment.

Whole-liver tissues were collected and fixed immediately after euthanasia. Fixation was performed in 5–10% formalin solution (prepared from 40% aqueous formaldehyde) for 7–10 days. After fixation, liver tissue fragments (~1 × 1 × 1.5 mm) were excised and subjected to dehydration through a graded ethanol series (40–100%).

Absolute ethanol was prepared from 96% ethanol using anhydrous copper sulfate (100 mL ethanol + 10 g CuSO_4_), with the mixture maintained for 1–2 days and density controlled using an alcohol meter. The samples were then processed through a two-step embedding procedure, including dehydration in absolute ethanol, treatment with chloroform, chloroform–paraffin mixture, and infiltration with pure paraffin.

Paraffin blocks were formed and sectioned using a microtome into 3–5 μm thick slices. For morphological evaluation, sections were deparaffinized and stained with hematoxylin and eosin (H&E). Histological evaluation was performed qualitatively by assessing hepatic architecture, hepatocellular degeneration, sinusoidal dilation, vascular congestion, and inflammatory infiltration. Hematoxylin stained basophilic structures (nuclei) blue-violet, while eosin stained eosinophilic components (cytoplasm and extracellular matrix) pink to red. Microphotographs were obtained using a light microscope at total magnifications up to ×800.

### 3.11. Antibacterial and Antifungal Activity

The antimicrobial and antifungal activities were evaluated according to the method described in [55]. The antimicrobial activity of the tested compounds was assessed against *Escherichia coli* ATCC 25922 B-RCM 0447, *Staphylococcus aureus* ATCC 6538 B-RCM 0470, *Klebsiella pneumoniae* B-RCM 0444, and *Pseudomonas aeruginosa* ATCC-9027 B-RCM 0476, while antifungal activity was evaluated against the yeast strain *Candida albicans* ATCC 885-653 Y-RCM 0475 obtained from the Republican Collection of Microorganisms (Astana, Kazakhstan). Ampicillin, penicillin, and nystatin were used as positive controls, while dimethyl sulfoxide (DMSO) was used as a negative control. Antimicrobial activity was evaluated using the agar well diffusion method, and the inhibition zones were measured in millimeters (mm), including the diameter of the well. In addition, the minimum inhibitory concentration (MIC, mg/mL) was determined using the microdilution method.

### 3.12. Statistical Analysis

Statistical analysis was performed using one-way analysis of variance (ANOVA) followed by Dunnett’s post hoc test for multiple comparisons versus the toxic control group. Data are presented as mean ± SD. Differences were considered statistically significant at *p* < 0.05.

## 4. Conclusions

This study demonstrated that the modification of 20-hydroxyecdysone through complexation with β-cyclodextrin leads to a significant enhancement in its biological activity. The modified derivatives included the Z-configured oxime 20-NOH, obtained by selective oximation of the C-6 carbonyl, and the triacetate 3Ac-20E, obtained by regioselective acetylation of the C-2, C-3, and C-22 hydroxyl groups. The obtained compounds are characterized by increased solubility, which enables multiple antioxidant mechanisms, including both free radical scavenging and reducing power, as confirmed by FRAP and DPPH assay results. In an experimental model of CCl_4_-induced liver injury, it was shown that the compounds under investigation exert a hepatoprotective effect, manifested by a reduction in transaminase activity (ALT, AST), a reduction in malondialdehyde levels, and improvement in the morphofunctional state of liver tissue, as confirmed by a comprehensive assessment of biochemical parameters, ultrasound and histological evaluations. It was shown that the enhancement in antioxidant activity is associated both with chemical modification of the molecule which alters the functional group profile and hydrogen-bonding capacity of the ecdysteroid scaffold and with the formation of inclusion complexes that improve the bioavailability of the active substance. At the same time, the antimicrobial activity of ecdysteroids remains moderate, with an MIC > 10 mg/mL for all bacterial strains tested, indicating weak antibacterial effects. The obtained results indicate the potential of β-cyclodextrin complexes of 20-hydroxyecdysone derivatives as promising antioxidant agents, which may be used in the development of new pharmaceutical preparations with hepatoprotective and antioxidant activity.

## Figures and Tables

**Table 1:** ^1^H and ^13^C chemical shifts of the substrate 3Ac-20E and β-cyclodextrin in the free state (δ_0_) and in the β-cyclodextrin inclusion complex (δ).

Atom No.	Group	δ_0_, ppm		δ, ppm		∆δ = δ − δ_0_	
		^1^H	^13^C	^1^H	^13^C	^1^H	^13^C
3-acetate of 20-hydroxyecdysone				Inclusion complex of 3-acetyl-20-hydroxyecdysone with β-cyclodextrin			
1_ax_ 1_eq_	CH_2_	1.65–1.70 m 2.11–2.22 m	37.19	1.65–1.70 m 2.11–2.20		0	
2	CH	4.95 д, ^3^*J* = 12.0 Hz	68.75	4.94 d		−0.01	
3	CH	5.17 br s	67.34	5.16		−0.01	
4_ax_ 4_eq_	CH_2_	1.65–1.70 m 1.90–1.93 m	31.41	1.65–1.70 1.90–1.92		0 −0.01	
5	CH	2.11–2.22 m	51.10	2.11–2.20		0.02	
6	>C=O	-	201.32	-			
7	CH	5.65 s	121.07	5.66		0.01	
8	>C<	-	165.83	-			
9	CH	3.02–3.08 m	34.09	3.02–3.08		0	
10	>C<	-	38.24				
11_ax_ 11_eq_	CH_2_	1.65–1.70 m 1.78–1.81 m	21.37	1.65–1.70 1.76–1.82		0 0.01	
12_ax_ 12_eq_	CH_2_	1.90–1.93 m 2.44–2.48	30.81	1.90–1.92 2.44–2.48		−0.01 0	
13	>C<	-	47.34	-			
14	>C<	-	83.51	-			
15_ax_ 15_eq_	CH_2_	1.91 s 2.11–2.22	33.52	1.90 s 2.11–2.20		−0.01 −0.02	
16_ax_ 16_eq_	CH_2_	2.11–2.22 m 2.44–2.48	21.70	2.11–2.20 2.44–2.48		−0.02 0	
17	CH	3.02–3.08	49.58	3.02–3.08		0	
18	-CH_3_	0.88 s	23.90	0.88 s		0	
19	-CH_3_	0.73 s	17.68	0.72 s		−0.01	
20	>C<	-	79.11	-			
21	-CH_3_	1.13 s	23.91	1.12 s		−0.01	
22	CH	4.63 d, ^3^*J* = 10.0 Hz	81.86	4.63 d		0	
23_ax_ 23_eq_	CH_2_	1.78–1.81 m 2.11–2.22 m	26.54	1.76–1.82 2.11–2.20		0.01 −0.02	
24_ax_ 24_eq_	CH_2_	1.78–1.81 m 2.11–2.22 m	40.63	1.76–1.82 2.11–2.20		0.01 −0.02	
25	>C<	-	75.24	-			
26	-CH_3_	1.28 s	26.29	1.27 s		−0.01	
27	-CH_3_	1.33 s	29.09	1.32 s		−0.01	
2	-OOC-CH_3_	1.88 s	170.31	1.87 s		−0.01	
3	-OOC-CH_3_	1.98 s	170.18	1.97 s		−0.01	
22	-OOC-CH_3_	2.05 s	172.92	2.04 s		−0.01	
β-Cyclodextrin							
1	CH	4.77 d, ^3^*J* = 4.0 Hz	102.40	4.78 d	102.45	0.01	0.05
2	CH	3.26 d, ^3^*J* = 12.1 Hz	72.83	3.26 d	72.92	0	0.09
3	CH	3.58 t, ^3^*J* = 8.3 Hz	73.54	3.60 t	73.56	0.02	0.02
4	CH	3.28 t, ^3^*J* = 10.0 Hz	81.98	3.28 t	82.03	0	0.05
5	CH	3.50 s	72.50	3.53 s	72.55	0.03	0.05
6	CH_2_	3.58 s	60.42	3.59 s	60.42	0.01	0
6	CH_2_	3.58 s	60.42	3.59 s	60.42	0.01	0

**Table 2:** Comparative ^1^H and ^13^C NMR data of the 20-hydroxyecdysone ketoxime and its inclusion complex with β-cyclodextrin.

Atom No.	Group	δ_0_ (^1^H), ppm	δ_0_ (^13^C), ppm	δ (^1^H), ppm (Complex)	δ (^13^C), ppm (Complex)	Δδ ^1^H	Δδ ^13^C
1ax/1eq	CH_2_	1.65–1.70 m/2.11–2.22 m	37.19	1.65–1.70 m/2.11–2.20 м	37.21	0	+0.02
2	CH	4.95 d (J = 12.0 Hz)	68.75	4.94 d	68.78	−0.01	+0.03
3	CH	5.17 br s	67.34	5.16 br s	67.36	−0.01	+0.02
4ax/4eq	CH_2_	1.65–1.70 m/1.90–1.93 m	31.41	1.65–1.70 m/1.90–1.92 m	31.42	0	+0.01
5	CH	2.11–2.22 m	36.0 *	2.11–2.20 m	36.1	−0.02	+0.1
6	C=N–OH	–	~154.0	–	~154.2	–	+0.2
7	CH	5.65 s	121.07	5.66 s	121.10	+0.01	+0.03
8	>C<	–	165.83	-	165.90	-	+0.07
9	CH	3.02–3.08 m	34.09	3.02–3.08 m	34.11	0	+0.02
10	>C<	–	38.24	–	38.30	-	+0.06
11ax/11eq	CH_2_	1.65–1.70 m/1.78–1.81 m	21.37	1.65–1.70 m/1.76–1.82 m	21.40	0	+0.03
12ax/12eq	CH_2_	1.90–1.93 m/2.44–2.48 m	30.81	1.90–1.92 m/2.44–2.48 m	30.83	−0.01	+0.02
13	>C<	–	47.34	–	47.38	–	+0.04
14	>C<	–	83.51	–	83.55	–	+0.04
15ax/15eq	CH_2_	1.91 s/2.11–2.22 m	33.52	1.90 s/2.11–2.20 m	33.55	−0.01	+0.03
16ax/16eq	CH	2.11–2.22 m/2.44–2.48 m	21.70	2.11–2.20 m/2.44–2.48 m	21.73	−0.02	+0.03
17	CH	3.02–3.08 m	49.58	3.02–3.08 m	49.60	0	+0.02
18	CH_3_	0.88 s	23.90	0.88 s	23.92	0	+0.02
19	CH_3_	0.73 s	17.68	0.72 s	17.70	−0.01	+0.02
20	>C<	–	79.11	–	79.15	–	+0.04
21	CH_3_	1.13 s	23.91	1.12 s	23.93	−0.01	+0.02
22	CH	4.63 d (J = 10.0 Hz)	81.86	4.63 d	81.90	0	+0.04
23ax/23eq	CH_2_	1.78–1.81 m/2.11–2.22 m	26.54	1.76–1.82 m/2.11–2.20 m	26.58	−0.02	+0.04
24ax/24eq	CH_2_	1.78–1.81 m/2.11–2.22 m	40.63	1.76–1.82 m/2.11–2.20 m	40.67	−0.02	+0.04
25	>C<	–	75.24	–	75.30	–	+0.06
26	CH_3_	1.28 s	26.29	1.27 s	26.31	−0.01	+0.02
27	CH_3_	1.33 s	29.09	1.32 s	29.12	−0.01	+0.03
β-Cyclodextrin-Based Inclusion Complexes							
Atom No.	Group	δ_0_ ^1^H, ppm	δ_0_ ^13^C, ppm	δ ^1^H, ppm	δ ^13^C, ppm	Δδ ^1^H	Δδ ^13^C
1	CH	4.77 d (J = 4.0 Hz)	102.40	4.78 d	102.45	+0.01	+0.05
2	CH	3.26 d (J = 12.1 Hz)	72.83	3.26 d	72.92	0	+0.09
3	CH	3.58 t (J = 8.3 Hz)	73.54	3.60 t	73.56	+0.02	+0.02
4	CH	3.28 t (J = 10.0 Hz)	81.98	3.28 t	82.03	0	+0.05
5	CH	3.50 s	72.50	3.53 s	72.55	+0.03	+0.05
6	CH_2_	3.58 s	60.42	3.59 s	60.42	+0.01	0

* The C-5 chemical shift is characteristic of the Z-oxime (syn orientation of the oxime OH group).

**Table 3:** Energies of the inclusion complexes (ONIOM [B3LYP/6-31G:UFF] method) (complexes are labeled according to Figure 3).

Parameters	Complex (a)	Complex (b)	Complex (c)	Complex (d)
Total energy, a.u.	−1636.472928	−1636.474272	−1636.470007	−2039.023082
ΔE, kj/mol	3.529	0.0	11.198	-
Stabilization energy, kj/mol	−38.668	−39.512	−36.836	−40.483
E_HOMO_, eV	−6.051	−6.058	−6.068	−6.440
E_LUMO_, eV	−0.934	−0.934	−0.951	−1.598

**Table 4:** Physicochemical characteristics of complexes (a–d) (ONIOM method [B3LYP/6-31G:UFF]).

Characteristic	Complex (a)	Complex (b)	Complex (c)	Complex (d)
E_0_, a.u.	2.088005	2.088232	2.085930	2.192096
μ, D	3.852	4.596	4.276	6.234
S, cal/(mol·K)	509.004	511.182	508.300	542.934
C_v_, cal/(mol·K)	404.053	403.195	402.358	429.919

**Table 5:** Concentration-dependent antioxidant activities of 20-hydroxyecdysone and its β-cyclodextrin inclusion complexes evaluated by FRAP and DPPH assays.

Concentration (mg/mL)	20E		20-NOH·β-CD		3Ac-20E·β-CD		Ascorbic Acid	BHA
	AAE/mL	% ±SD	AAE/mL	% ±SD	AAE/mL	% ±SD	AAE/mL	% ±SD
0.05	0.451 ± 0.037	0.42 ± 1.18	1.304 ± 0.525	91.57 ± 2.09	0.580 ± 0.25	88.82 ± 8.55	0.45 ± 0.02	79.8 ± 0.6
0.10	0.415 ± 0.018	1.35 ± 1.18	1.755 ± 0.595	93.09 ± 2.09	0.44 ± 0.26	93.28 ± 8.55	0.90 ± 0.03	80.3 ± 0.5
0.15	0.441 ± 0.008	0.45 ± 1.08	1.528 ± 0.595	93.46 ± 0.25	0.88 ± 0.26	93.07 ± 0.38	1.35 ± 0.04	80.6 ± 0.4
0.20	0.460 ± 0.021	0.45 ± 1.18	1.304 ± 0.595	93.46 ± 2.09	0.58 ± 0.26	93.07 ± 8.55	1.80 ± 0.04	80.8 ± 0.4
0.25	0.065 ± 0.004	1.43 ± 1.18	2.852 ± 0.520	93.36 ± 2.09	3.76 ± 0.06	93.34 ± 8.55	1.93 ± 0.05	80.7 ± 0.5
0.50	0.069 ± 0.014	66.00 ± 2.63	2.539 ± 0.520	95.94 ± 0.10	3.70 ± 0.06	95.75 ± 0.42	2.28 ± 0.06	80.5 ± 0.5
0.75	0.054 ± 0.004	73.75 ± 2.63	3.452 ± 0.520	94.42 ± 0.10	3.82 ± 0.06	95.52 ± 0.42	2.26 ± 0.06	80.6 ± 0.5
1.00	0.139 ± 0.080	79.14 ± 1.00	2.829 ± 0.124	94.66 ± 0.53	3.81 ± 0.28	95.86 ± 0.29	2.32 ± 0.07	80.7 ± 0.5

**Table 6:** Biochemical parameters following CCl_4_ exposure and hepatoprotection by studied compounds.

Group	Dose, mg/kg	ALT (U/L)	AST (U/L)	De Ritis Ratio	MDA (µmol/L)
Intact	-	135.6 ± 0.5 *	71.9 ± 0.1 *	0.53	0.55 ± 0.01 *
Control	-	153.6 ± 0.3	79.9 ± 0.2	0.52	0.60 ± 0.01
Carsil	-	121.4 ± 0.4 *	73.7 ± 0.3 *	0.61	0.57 ± 0.01 *
20E	25	132.5 ± 0.5 *	60.8 ± 0.2 *	0.46	1.07 ± 0.02 *
	50	114.7 ± 0.4 *	73.8 ± 0.2 *	0.64	1.51 ± 0.01 *
	75	106.7 ± 0.2 *	76.8 ± 0.3 *	0.61	1.82 ± 0.01 *
20-NOH·β-CD	25	97.7 ± 0.1 *	71.6 ± 0.3 *	0.73	0.07 ± 0.01 *
	50	116.7 ± 0.2 *	61.7 ± 0.2 *	0.53	0.92 ± 0.01 *
	75	179.7 ± 0.4	62.5 ± 0.3 *	0.35	3.24 ± 0.01
3Ac-20E·β-CD	25	224.3 ± 0.4	49.8 ± 0.3 *	0.22	0.82 ± 0.01 *
	50	126.9 ± 0.1 *	62.5 ± 0.3 *	0.49	1.68 ± 0.01 *
	75	116.9 ± 1.6 *	66.5 ± 0.3 *	0.57	1.93 ± 0.01 *

*—Statistically significant difference compared to toxic control group (one-way ANOVA followed by Dunnett’s post hoc test, *p* < 0.05). Data are expressed as mean ± SD. Each experimental group included 4 rats (*n* = 4).

**Table 7:** Antimicrobial and antifungal activity of 20E and its derivatives.

Microorganisms Tested	Compounds			Positive Control		
	20E, mm	20-NOH·β-CD, mm	3Ac-20E·β-CD, mm	Penicillin, mm	Ampicillin, mm	Nystatin, Mm
*E. coli*	23.7 ± 1.5	17.7 ± 2.1	-	-	-	-
*S. aureus*	27.0 ± 2.0	15.3 ± 0.6	-	28.6 ± 0.85	-	-
*K. pneumoniae*	22.7 ± 2.9	16.3 ± 1.5	-	-	-	-
*P. aeruginosa*	24.3 ± 2.1	16.3 ± 2.9	12.7 ± 0.6	-	15.9 ± 0.25	-
*C. albicans*	-	13.7 ± 2.9	13.2 ± 1.0	-	-	14.5 ± 0.70

>20 mm strong activity, 10–20 mm moderate activity, and <10 mm weak or no activity.

## Data Availability

The original contributions presented in this study are included in the article. Further inquiries can be directed to the corresponding authors.

## Supplementary Materials

The following supporting information can be downloaded at: https://www.mdpi.com/article/10.3390/ph19060885/s1, Figure S1: ^1^H NMR Spectrum of 20-NOH·β-CD (500 MHz, DMSO-d6); Figure S2: ^13^C NMR Spectrum of 20-NOH·β-CD (125 MHz, DMSO-d6).

## Abbreviations

The following abbreviations are used in this manuscript:

20E	20-hydroxyecdysone (ecdysterone);
20-NOH	ketoxime derivative of 20-hydroxyecdysone;
20-NOH·β-CD	inclusion complex of the ketoxime derivative of 20-hydroxyecdysone with β-cyclodextrin;
3Ac-20E	2,3,22-triacetate of 20-hydroxyecdysone
3Ac-20E·β-CD	inclusion complex of the 2,3,22-triacetate derivative of 20-hydroxyecdysone with β-cyclodextrin;
AAE	ascorbic acid equivalents;
ALT	alanine aminotransferase;
AST	aspartate aminotransferase;
BHA	butylated hydroxyanisole;
β-CD	β-cyclodextrin;
CCl_4_	carbon tetrachloride;
CDCl_3_	deuterated chloroform;
DMSO	dimethyl sulfoxide;
DMSO-d_6_	deuterated dimethyl sulfoxide;
DPPH	2,2-diphenyl-1-picrylhydrazyl;
EC_50_	half-maximal effective concentration;
FRAP	ferric reducing antioxidant power;
H&E	hematoxylin and eosin;
HPLC	high-performance liquid chromatography;
IC_50_	half-maximal inhibitory concentration;
LPO	lipid peroxidation;
MDA	malondialdehyde;
MIC	minimum inhibitory concentration;
NMR	nuclear magnetic resonance;
ONIOM	our own N-layered integrated molecular orbital and molecular mechanics;
PM6	parameterized model 6 (semiempirical method);
SD	standard deviation;
UFF	universal force field;
UV-Vis	ultraviolet–visible spectroscopy.
