# Supplementary material for: β-Cyclodextrin Inclusion Complexes of 20-Hydroxyecdysone Derivatives: Synthesis, NMR Characterization, and In Vitro/In Vivo Evaluation of Antioxidant, Hepatoprotective, and Antimicrobial Activities
Source: Pharmaceuticals (Basel). 2026 Jun 2;19(6):885. doi: 10.3390/ph19060885 (PMC13304566; doi:10.3390/ph19060885)
Supplement: Supplementary file 1 [file pharmaceuticals-19-00885-s001.zip › pharmaceuticals-4284223-supplementary.pdf]

Figure S1. 1H NMR Spectrum of 20-NOH·β-CD (500 MHz, DMSO-d6)

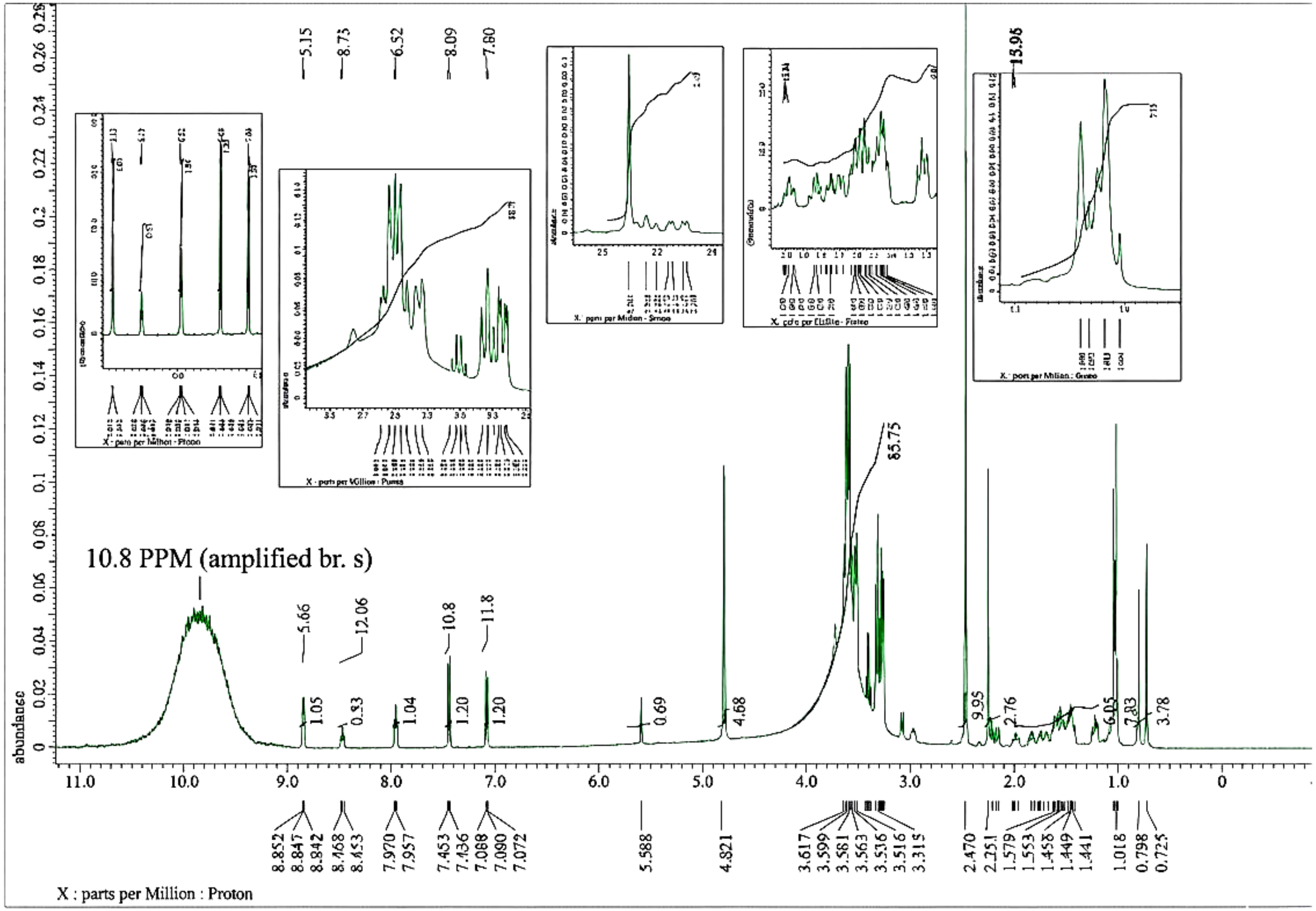

δ 10.8 ppm (1H, br s, N–OH); 5.66 ppm (1H, d, J = 2.6 Hz, H-7); 0.77–1.09 ppm (15H, 5s, 5xCH3).

Figure S2. 13C NMR Spectrum of 20-NOH·β-CD (125 MHz, DMSO-d6)

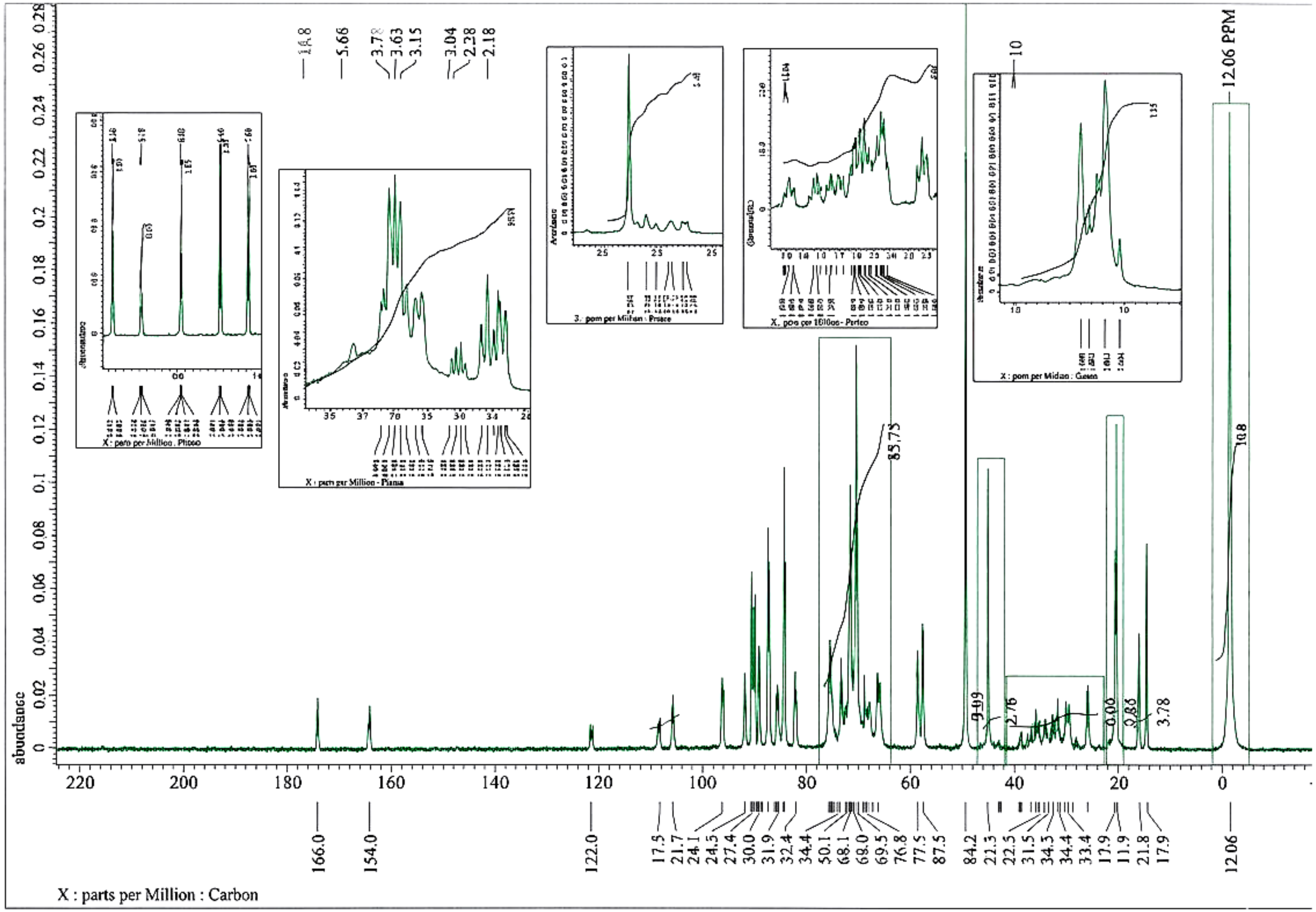

δ 154.0 ppm (C-6, C=N–OH); 166.0 ppm (C-8); 36.0 ppm (C-5, Z-configuration). Absence of C-6 ketone at 203.4 ppm confirms oxime formation.
